# Advancements in Chitosan-Based Scaffolds for Chondrogenic Differentiation and Knee Cartilage Regeneration: Current Trends and Future Perspectives

**DOI:** 10.3390/bioengineering12070740

**Published:** 2025-07-07

**Authors:** Kamila Rawojć, Ryszard Tadeusiewicz, Ewa Zych-Stodolak

**Affiliations:** 1 Department of Biocybernetics and Biomedical Engineering, AGH University of Science and Technology, 30-059 Krakow, Poland; 2 Department of Biomaterials and Composites, Faculty of Materials Science and Ceramics, AGH University of Science and Technology, 30-059 Krakow, Poland

**Keywords:** chitosan scaffolds, cartilage regeneration, chondrogenic differentiation, tissue engineering, mesenchymal stem cells, knee joint repair

## Abstract

Cartilage damage, particularly in the knee joint, presents a significant challenge in regenerative medicine due to its limited capacity for self-repair. Conventional treatments like microfracture surgery, autologous chondrocyte implantation (ACI), and osteochondral allografts often fall short, particularly in cases of larger defects or degenerative conditions. This has led to a growing interest in tissue engineering approaches that utilize biomaterial scaffolds to support cartilage regeneration. Among the many materials explored, chitosan—a naturally derived polysaccharide—has gained attention for its biocompatibility, biodegradability, and structural resemblance to the extracellular matrix (ECM) of cartilage. Recent advances in scaffold design have focused on modifying chitosan to improve its mechanical properties and enhance its biological performance. These modifications include chemical crosslinking, the incorporation of bioactive molecules, and the development of composite formulations. Such enhancements have allowed chitosan-based scaffolds to better support mesenchymal stem cell (MSC) differentiation into chondrocytes, paving the way for improved regenerative strategies. This review explores the latest progress in chitosan scaffold fabrication, preclinical findings, and the transition toward clinical applications. It also discusses the challenges that need to be addressed, such as mechanical stability, degradation rates, and the successful translation of research into viable therapeutic solutions.

## 1. Introduction

Articular cartilage is a highly specialized connective tissue that plays a fundamental role in joint biomechanics by providing a low-friction surface for articulation and absorbing mechanical loads. Its unique extracellular matrix (ECM), composed predominantly of type II collagen and proteoglycans rich in glycosaminoglycans (GAGs), ensures both tensile strength and compressive resistance. However, articular cartilage is avascular, aneural, and lacks lymphatic drainage, severely limiting its intrinsic regenerative capacity following trauma or degenerative disease such as osteoarthritis. Consequently, even minor focal lesions often fail to heal and may progress to joint degeneration, pain, and disability if left untreated [1,2].

Conventional therapeutic strategies such as microfracture surgery, autologous chondrocyte implantation (ACI), mosaicplasty, and osteochondral allografts aim to restore cartilage function but are associated with significant limitations. These include fibrocartilage formation instead of hyaline cartilage, donor site morbidity, risk of immunogenic reactions, and limited long-term efficacy, particularly in cases involving large or deep defects [3,4]. The suboptimal clinical outcomes of these methods have prompted a paradigm shift toward regenerative medicine and tissue engineering, which seek to replicate the biological, structural, and functional attributes of native cartilage through a combination of biomaterials, cells, and bioactive molecules.

Among the myriad of biomaterials investigated for cartilage tissue engineering, chitosan—a linear polysaccharide derived from the deacetylation of chitin—has emerged as a particularly promising scaffold component. Its chemical structure bears a striking resemblance to natural GAGs, enabling it to interact favorably with ECM components and cellular receptors [5]. Chitosan is biodegradable, non-toxic, and demonstrates intrinsic antimicrobial and hemostatic properties. Its cationic nature facilitates electrostatic interactions with anionic cell membranes and ECM proteins, enhancing cell adhesion, proliferation, and phenotypic maintenance [6,7].

Recent technological advancements have enabled the precise manipulation of chitosan’s molecular weight, degree of deacetylation, and physicochemical configuration. These modifications allow the tuning of scaffold stiffness, porosity, and degradation rate to meet specific requirements for chondrogenic differentiation and in vivo integration [8]. Moreover, chitosan can be processed into diverse forms—gels, sponges, membranes, and nanofibers—using techniques such as freeze-drying, solvent casting, and electrospinning. Notably, electrospun chitosan nanofibers have demonstrated high surface area-to-volume ratios and mechanical features favorable for guiding mesenchymal stem cell (MSC) differentiation and ECM deposition [9,10].

Emerging composite systems have further expanded the functionality of chitosan-based scaffolds. Hybrid formulations incorporating chondroitin sulfate, hyaluronic acid, or collagen enhance the biological mimicry of cartilage ECM, while the inclusion of nano-hydroxyapatite or magnesium ions augments bioactivity and osteochondral integration [11,12]. Innovative drug-loaded variants, such as norfloxacin- or ursolic acid-containing chitosan scaffolds, have also demonstrated antimicrobial and anti-inflammatory properties, which are crucial in post-operative healing and the prevention of infection in cartilage repair procedures [13,14].

In addition to material design, efforts are underway to optimize scaffold bioactivity via the incorporation of signaling molecules such as TGF-β1, BMPs, IGF-1, and FGF-2, which are known to drive chondrogenesis. Scaffold-mediated delivery systems provide sustained, localized release of these factors, enabling spatial and temporal control over MSC differentiation pathways [15]. Furthermore, the functionalization of scaffold surfaces with peptides like RGD or fibronectin domains has been shown to significantly improve stem cell adhesion and cytoskeletal organization, thus supporting long-term matrix production [16].

Despite promising in vitro and preclinical results, challenges remain in clinical translation. These include variability in scaffold manufacturing, batch-to-batch reproducibility, immunogenicity associated with animal-derived chitosan sources, and insufficient mechanical performance under physiological loading conditions. Regulatory hurdles, especially those concerning sterilization, bioburden control, and Good Manufacturing Practice (GMP) compliance, also pose barriers to commercialization [17]. Addressing these challenges will require interdisciplinary approaches bridging materials science, cellular biology, biomechanics, and clinical orthopedics.

The aim of this review is to provide a critical and comprehensive analysis of the current landscape of chitosan-based scaffolds in cartilage tissue engineering, with a focus on scaffold design, biofunctionalization strategies, in vitro and in vivo outcomes, and translational challenges. We highlight recent innovations that are redefining the potential of chitosan as a cornerstone material in the regenerative repair of knee cartilage defects and beyond.

## 2. Chitosan Scaffolds in Cartilage Tissue Engineering

Chitosan, a deacetylated derivative of chitin sourced predominantly from crustacean shells, represents one of the most versatile and biologically favorable polysaccharides for use in cartilage tissue engineering. Its structural similarity to naturally occurring glycosaminoglycans (GAGs) in the cartilage extracellular matrix (ECM) allows for functional mimicry at both biochemical and biomechanical levels [7]. This resemblance supports cellular adhesion, proliferation, and phenotype retention, making chitosan a leading candidate for scaffold-based strategies in cartilage repair [5].

From a physicochemical standpoint, chitosan possesses several properties ideal for scaffold fabrication. It is positively charged under physiological conditions, which promotes interaction with negatively charged cell membranes and ECM proteins. Furthermore, it is biodegradable via lysozyme-mediated hydrolysis and biocompatible, eliciting minimal immunogenic response in most in vivo models [6]. Its solubility in mildly acidic aqueous solutions enables processing into a variety of scaffold forms including porous sponges, hydrogels, and nanofibrous mats, thereby offering structural flexibility suitable for diverse clinical indications [13].

The porosity and architecture of chitosan-based scaffolds are central to their function in cartilage engineering. Scaffolds fabricated using freeze-drying techniques can exhibit pore sizes in the optimal range (100–300 µm) for chondrocyte infiltration and nutrient diffusion [13]. Moreover, electrospinning has enabled the production of nanofibrous scaffolds with diameters approximating collagen fibrils, thereby enhancing biomimicry and supporting chondrogenic differentiation of mesenchymal stem cells (MSCs) [9]. The incorporation of biofunctional groups or bioactive molecules such as RGD peptides or fibronectin domains further improves cellular attachment and modulates intracellular signaling cascades essential for chondrogenesis [16].

Aside from physical modifications, the integration of chitosan with other polymers and bioactive fillers has led to significant advances in scaffold performance. Composite scaffolds combining chitosan with collagen, gelatin, or synthetic polymers such as polyvinyl alcohol (PVA) or polylactic acid (PLA) exhibit enhanced mechanical strength and improved viscoelastic behavior, necessary for load-bearing applications in articulating joints [18]. Of particular interest are blends incorporating nano-hydroxyapatite or magnesium-doped hydroxyapatite, which contribute osteoconductivity and modulate ion release to promote osteochondral interface regeneration [14].

Biological enhancement of chitosan scaffolds can also be achieved via encapsulation or surface tethering of growth factors such as transforming growth factor-beta 1 (TGF-β1), insulin-like growth factor (IGF-1), or bone morphogenetic proteins (BMPs). These growth factors, when delivered locally and in a controlled-release fashion, significantly enhance MSC recruitment and drive their differentiation into chondrocytes [15]. Additionally, the co-delivery of anti-inflammatory agents such as ursolic acid has been shown to modulate macrophage phenotype toward an M2 reparative profile, potentially accelerating tissue regeneration and integration [14].

The functional validation of chitosan-based scaffolds has been demonstrated in numerous in vitro and in vivo studies. For instance, Ho et al. reported enhanced osteoblast proliferation and maturation on chitosan nanofibers, indicating their potential for osteochondral regeneration [9]. In another study, Sancho-Tello et al. showed that the addition of platelet-rich plasma (PRP) to porous chitosan scaffolds significantly increased type II collagen expression in cultured human chondrocytes, supporting phenotypic maturation [17]. Furthermore, dental pulp stem cells seeded onto chitosan scaffolds modified with RGD or fibronectin displayed improved spreading, viability, and cytoskeletal organization, highlighting the importance of microenvironmental cues in stem cell differentiation [16].

Importantly, degradation kinetics of chitosan must be balanced with neotissue formation to avoid premature scaffold resorption or prolonged foreign body response. The degradation rate can be tuned via degree of deacetylation, crosslinking strategies (e.g., genipin, glutaraldehyde), or polymer blending. The optimization of these parameters is essential to ensure scaffold persistence throughout the critical early stages of cartilage remodeling [8].

In summary, chitosan-based scaffolds provide a flexible and bioactive platform for cartilage regeneration. Advances in scaffold design, composite formation, and growth factor delivery have significantly enhanced their biological performance. Continued innovation is likely to focus on integrative strategies that combine biomechanical support with immunomodulation, controlled drug release, and multiscale structural mimicry of native cartilage.

## 3. Strategies for Scaffold Biofunctionalization

While the physicochemical properties of chitosan provide a strong foundation for tissue engineering applications, the functional success of a scaffold in cartilage regeneration is largely determined by its bioactivity—that is, its ability to modulate cellular behavior through biochemical and biophysical cues. Biofunctionalization strategies aim to enhance this bioactivity by modifying the scaffold surface or bulk material to actively influence cell adhesion, proliferation, differentiation, and matrix synthesis. Such modifications are particularly crucial in chondral tissue engineering, where the scaffold must not only support chondrocyte or progenitor cell survival but should also direct the formation of a stable cartilaginous extracellular matrix under mechanically active conditions.

One of the most effective and widely used approaches involves the incorporation of bioactive peptides into the chitosan scaffold, most notably the arginine–glycine–aspartic acid (RGD) motif. This tri-peptide sequence, found in fibronectin and other ECM proteins, binds integrin receptors on the cell surface and initiates intracellular signaling cascades that regulate cell spreading and focal adhesion formation [19]. The covalent immobilization of RGD on chitosan scaffolds has been shown to significantly enhance stem cell adhesion and viability, particularly for human dental pulp stem cells and human MSCs, which otherwise form spheroids on unmodified chitosan due to its relatively poor cell-adhesive surface chemistry [20].

Alternatively, adsorption or covalent tethering of ECM proteins, such as fibronectin, laminin, or collagen, offers another pathway for enhancing cell–scaffold interaction. Asghari Sana et al. demonstrated that fibronectin-functionalized chitosan scaffolds supported the significantly improved attachment and cytoskeletal organization of hDPSCs compared to unmodified controls [21]. These findings underscore the importance of tailoring the scaffold’s biochemical microenvironment to better recapitulate the native cartilage niche.

Another prominent strategy involves growth factor incorporation, either via encapsulation within the scaffold matrix or surface immobilization. Chitosan’s chemical structure enables ionic interactions with a wide array of proteins, making it an effective carrier for molecules such as TGF-β1, IGF-1, BMP-2, and FGF-2—all of which are implicated in chondrogenic differentiation [21]. Controlled-release formulations allow the localized, sustained delivery of these signals to cells seeded within or recruited into the scaffold. Recent advances in microencapsulation techniques, such as polyelectrolyte complex nanoparticles, have improved the temporal control of growth factor availability, thus promoting effective lineage commitment of MSCs [22].

Furthermore, biofunctionalization strategies have been extended to include immunomodulatory agents, which help to steer the post-implantation immune response toward a regenerative phenotype. For example, the integration of ursolic acid, a natural pentacyclic triterpenoid with anti-inflammatory and osteoinductive properties, into mesoporous hydroxyapatite-chitosan scaffolds has been shown to promote M2 macrophage polarization and upregulate osteochondral markers such as BMP-2, RUNX2, and COL1 in vitro and in vivo [14]. This dual osteo-immunomodulatory function positions such scaffolds as promising candidates for osteochondral defect repair.

Physical strategies have also gained momentum, particularly the nanotopographical modification of scaffold surfaces. Electrospun nanofibers and microgrooved surfaces have been shown to influence cell orientation, migration, and gene expression. These topographical cues synergize with biochemical signals to enhance matrix deposition and anisotropy, both of which are essential for recreating the zonal architecture of native articular cartilage [23]. Additionally, mechanical preconditioning in bioreactors simulating joint loading can further amplify chondrogenic signaling in stem cells and improve the mechanical properties of the resulting neo-cartilage [24].

Recent advancements have introduced mechanically responsive chitosan scaffolds that alter their properties under applied stress, enabling dynamic performance in load-bearing environments. One fabrication strategy involves thermally induced phase separation (TIPS) followed by freeze-gelation and photo-crosslinking, which produces scaffolds with enhanced compressive strength and elastic recovery—capable of withstanding multiple compression cycles (≈80% recovery over five cycles) without toxic crosslinkers [24]. Another approach engineers high-elasticity chitosan matrices through hydrogen-bond modulation techniques, allowing scaffolds to be compressed for minimally invasive delivery and then to fully recover their original shape—maintaining structural integrity even after 100 compressive cycles [25]. Beyond bulk elasticity, specialized mechano-sensitive systems incorporate microcapsules or microstructures that release bioactive agents (e.g., TGF-β3) in response to mechanical loading or oscillatory fluid flow, as demonstrated in bone regeneration models [26]. While promising, these mechano-responsive systems remain primarily at the in vitro stage, highlighting the need for the further optimization of loading parameters (frequency, amplitude, duration) to fully harness mechanical stimuli for scaffold performance in vivo.

Lastly, smart scaffold platforms—capable of responding to environmental stimuli (pH, enzymes, mechanical stress)—are emerging as a frontier in scaffold biofunctionalization. These dynamic systems can modulate drug release or scaffold stiffness in situ, allowing adaptive responses to the changing tissue microenvironment. Chitosan’s responsive nature, especially its pH sensitivity and enzymatic degradability, makes it an attractive backbone for designing such intelligent systems [27,28].

**Figure 1:**
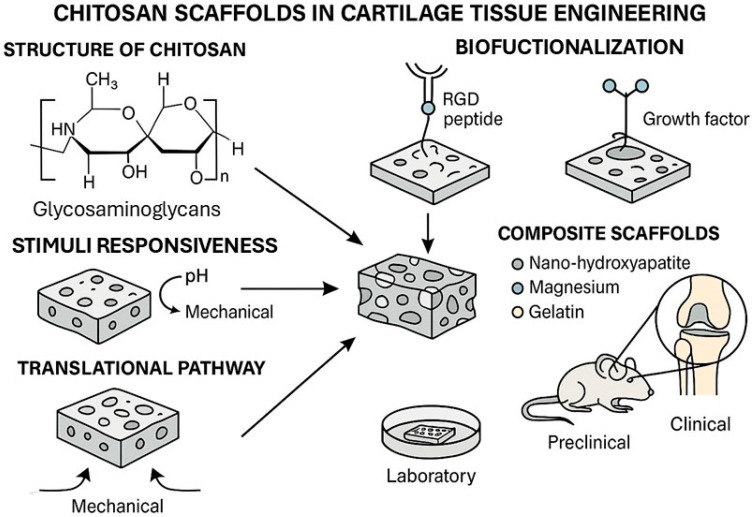
A schematic representation of smart chitosan-based scaffold systems for cartilage regeneration.

Figure 1 illustrates the platform that responds to environmental stimuli such as pH changes, enzymatic activity, and mechanical loading, triggering the localized release of therapeutic proteins or growth factors and promoting targeted cellular responses. This dynamic behavior enhances tissue integration, modulates inflammation, and supports chondrogenic differentiation in situ.

Figure 1 illustrates the platform that responds to environmental stimuli such as pH changes, enzymatic activity, and mechanical loading, triggering localized release of therapeutic proteins or growth factors and promoting targeted cellular responses. This dynamic behavior enhances tissue integration, modulates inflammation, and supports chondrogenic differentiation in situ.

In summary, scaffold biofunctionalization strategies—from biochemical ligand attachment and growth factor delivery to immunomodulation and biomechanical conditioning—are critical to optimizing the regenerative capacity of chitosan-based systems. The integration of multiple biofunctionalization modalities within a single scaffold represents the future direction of this field, aiming to emulate the complexity of native cartilage and to meet the rigorous demands of clinical application.

## 4. Material and Functional Enhancements in Chitosan-Based Scaffolds for Cartilage Regeneration

The regenerative potential of chitosan-based scaffolds is not only dependent on their innate biocompatibility and biodegradability, but also on their tunable physical, chemical, and structural properties that can be engineered to match specific regenerative requirements. The source, molecular weight, degree of deacetylation (DD), and processing technique of chitosan substantially influence its mechanical integrity, degradation rate, porosity, and, most critically, its interaction with stem cells and native tissue [8].

Recent research highlights that medical-grade chitosan with a high degree of deacetylation (≥92.6%) offers markedly superior biological performance compared to conventional, lower-DD variants. In a 2022 study, Yurtsever et al. demonstrated that rat adipose-derived mesenchymal stem cells (rAdMSCs) exhibited significantly enhanced attachment, proliferation, and cytoskeletal organization on high-DD medical-grade chitosan scaffolds. In contrast, control scaffolds with lower DD (75–85%) induced spheroid formation and hindered cell spreading—an effect detrimental to cartilage regeneration [29]. Furthermore, genipin crosslinking, when applied judiciously, contributed to a reduction in pore size and water uptake without compromising the interconnected porosity necessary for cell migration and nutrient diffusion.

Micro-CT and SEM analyses confirmed that medical-grade scaffolds possess a highly interconnected and homogeneous pore structure, with pore sizes in the range of 100–200 µm, which is optimal for cartilage cell ingrowth. Mechanical properties and hydrophilicity were effectively modulated via glycerol phosphate and genipin crosslinking, supporting prolonged scaffold integrity and biomolecule retention under physiological conditions [30].

To further augment the regenerative microenvironment, piezoelectric modifications of chitosan-based scaffolds have emerged as a cutting-edge strategy, particularly in systems where mechanical loading is integral to healing, such as the knee joint. Wu et al. recently engineered a chitosan/gelatin-based piezoelectric scaffold incorporating polydopamine-functionalized hydroxyapatite and barium titanate nanoparticles (PDA-HA/PBT), demonstrating not only improved mechanical stiffness and electrical output but also immunomodulatory and pro-angiogenic activity [31]. These piezoelectric hydrogels promoted M2 macrophage polarization and enhanced VEGF and BMP-2 expression both in vitro and in a rat cranial defect model. Transcriptome analysis revealed activation of the PI3K/Akt signaling pathway, suggesting a mechanoelectrical mechanism of immune modulation and tissue repair.

Such bioelectrically active scaffolds hold promise for load-bearing cartilage interfaces, where mechanical forces may be transduced into regenerative signals, mimicking the intrinsic piezoelectric properties of native bone and cartilage [32]. When combined with biochemical cues and optimized porosity, these systems may significantly accelerate the repair of osteochondral defects by synchronizing the phases of inflammation, angiogenesis, and chondrogenesis.

In addition, scaffold customization through 3D fabrication techniques such as freeze-drying and electrospinning enables the production of layered or gradient scaffolds that mimic the zonal architecture of native cartilage. Multiphase constructs that replicate the superficial, transitional, and calcified cartilage zones—or that span cartilage–bone interfaces—have shown enhanced cellular zonation and ECM deposition in vitro, offering translational relevance for large or complex defects [33].

Altogether, chitosan’s versatility as a structural and bioactive platform is greatly amplified by advanced material engineering approaches. Integrating high-purity formulations, targeted crosslinking, and electroactive enhancements, next-generation chitosan scaffolds can be tailored to meet the complex demands of personalized, load-bearing cartilage regeneration.

## 5. In Vivo Applications and Translational Challenges of Chitosan-Based Scaffolds

### 5.1. In Vivo Applications

Chitosan-based scaffolds have demonstrated notable regenerative capacity in vivo, particularly in preclinical models targeting osteochondral defects. High-purity, medical-grade chitosan with a high degree of deacetylation (≥92%) has proven superior to lower-grade variants in supporting stem cell adhesion, proliferation, and extracellular matrix (ECM) synthesis. For example, Yurtsever et al. demonstrated that rat adipose-derived mesenchymal stem cells (rAdMSCs) cultured on high-DD chitosan scaffolds exhibited increased actin cytoskeletal organization and ECM protein expression compared to standard scaffold formulations [29].

Composite chitosan-based biomaterials have also shown excellent regenerative potential. In a rat model of osteochondral injury, scaffolds incorporating platelet-rich plasma (PRP) enhanced chondrogenic differentiation and stimulated hyaline-like cartilage formation, supported by type II collagen and proteoglycan expression [17]. Furthermore, the incorporation of immunomodulatory agents such as ursolic acid into mesoporous hydroxyapatite/chitosan scaffolds has promoted M2 macrophage polarization and improved bone–cartilage interface healing in vivo [14].

Emerging piezoelectric composites integrating polydopamine-functionalized hydroxyapatite and barium titanate (PDA-HA/PBT) into gelatin/chitosan matrices have also demonstrated promise. Wu et al. reported that these smart scaffolds enhanced angiogenesis, osteoinduction, and immune modulation in rat calvarial models through mechanotransduction mechanisms involving the PI3K/Akt pathway [30]. Most in vivo studies remain preclinical, their outcomes validate chitosan’s performance across biological, structural, and immunological dimensions, setting the stage for well-designed human trials.

### 5.2. Translational Challenges

Despite these compelling findings, chitosan scaffolds face several translational barriers. First, variability in source material (e.g., crustacean shell, fungal origin), molecular weight, and degree of deacetylation continues to complicate standardization and quality assurance, which is crucial for regulatory acceptance [6]. Batch-to-batch inconsistencies in scaffold porosity, degradation rate, and mechanical strength can affect reproducibility and efficacy across patient cohorts.

Secondly, manufacturing scale-up under GMP conditions remains a bottleneck, particularly for scaffolds incorporating bioactive agents or multilayered architectures. Additionally, regulatory classification ambiguity (device vs. biologic vs. combination product) may impose complex and divergent approval pathways across different regulatory bodies such as the FDA, EMA, or PMDA [34].

Moreover, long-term in vivo studies addressing biodegradation kinetics, chronic inflammatory responses, and scaffold–host integration are limited. Post-marketing surveillance data are sparse, further complicating cost–benefit assessments needed for clinical reimbursement and guideline adoption.

An important yet often overlooked factor contributing to the translational limitations of chitosan-based scaffolds is the source of the parent chitin, which can significantly affect the immunological profile of the final biomaterial. Most commercially available chitosan is derived from crustacean shells (e.g., shrimp, crab), which may contain residual protein contaminants or endotoxins capable of eliciting unwanted innate immune activation, especially in sensitive or allergic individuals. Studies have demonstrated that the insufficient purification of chitosan can lead to elevated levels of Toll-like receptor (TLR) activation, increased cytokine production (e.g., IL-6, TNF-α), and macrophage polarization toward a pro-inflammatory M1 phenotype [34]. In contrast, fungal-derived chitosan exhibits a lower immunogenic profile due to its reduced protein content and lower endotoxin burden [35]. Moreover, fungal chitosan presents advantages for GMP compliance and scalability, offering a more consistent source material that aligns better with regulatory expectations for medical-grade scaffolds. Therefore, the careful selection of the chitin source and implementation of stringent purification protocols are essential for minimizing immunological risk and enhancing the safety profile of chitosan-based constructs intended for clinical use [36,37].

Addressing these translational challenges will require interdisciplinary collaboration between biomaterial scientists, bioengineers, clinicians, and regulatory experts to ensure clinical-grade chitosan scaffolds meet the stringent demands of modern regenerative orthopedics.

### 5.3. Clinical Landscape Overview

Table 1 summarizes recent and ongoing clinical and advanced preclinical trials initiated since 2020 that involve chitosan-based systems for cartilage regeneration. These studies span injectable carboxymethyl-chitosan hydrogel formulations for osteoarthritis, composite scaffolds functionalized with immunomodulatory exosomes, and bioactive peptides promoting cartilage-specific lineage commitment.

Although most trials remain in Phase I or are restricted to preclinical animal models, the expanding diversity in design and therapeutic targets reflects growing confidence in chitosan’s translational potential. Nevertheless, regulatory readiness, consistent clinical endpoints, and real-world efficacy data remain necessary prerequisites for widespread clinical deployment.

**Figure 2:**
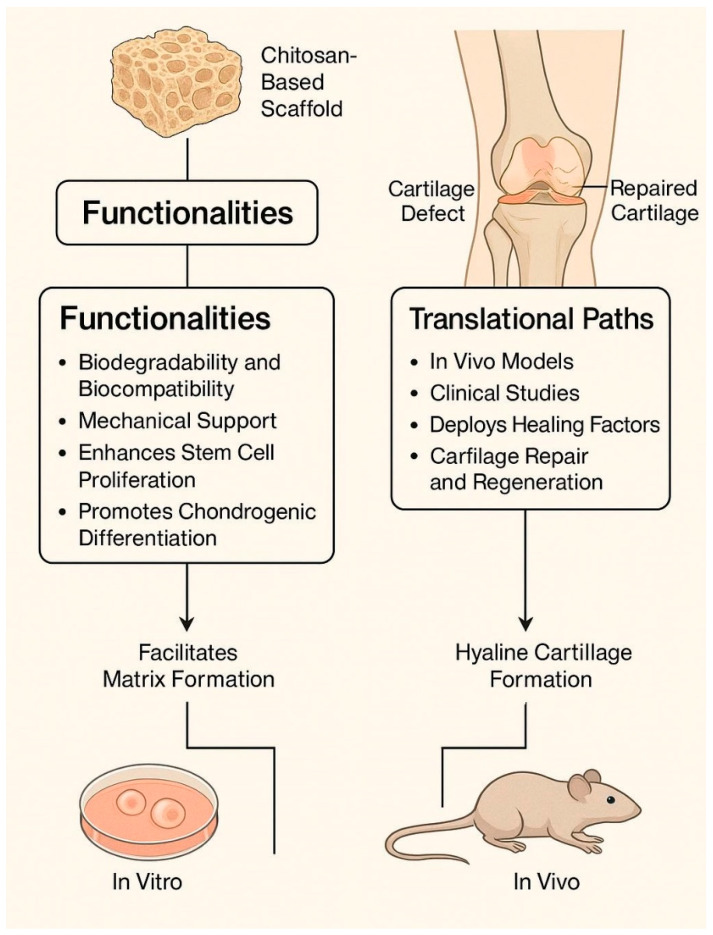
An overview of chitosan-based scaffold functionalities and translational pathways.

Figure 2 summarizes the core biological functionalities, structural properties, and therapeutic applications of chitosan-based scaffolds in cartilage regeneration. It also outlines the critical steps in translational development, from laboratory fabrication through preclinical validation to clinical implementation.

## 6. Future Perspectives: Next-Generation Chitosan Scaffolds and Emerging Technologies

The field of cartilage tissue engineering is rapidly evolving toward multifunctional, intelligent, and patient-specific scaffold systems. Chitosan, as a versatile and tunable biopolymer, is well-positioned to serve as the foundational platform for these next-generation regenerative technologies. However, to fully unlock its clinical potential, future innovations must address not only the biomaterial’s biological performance but also its ability to adapt dynamically to complex in vivo environments and personalized therapeutic needs.

One of the most promising frontiers lies in the integration of smart or stimuli-responsive systems into chitosan scaffolds. Such materials can respond to environmental triggers—pH, temperature, enzymatic activity, or mechanical stress—to modulate drug release, scaffold stiffness, or degradation rate. Thermoresponsive chitosan-based hydrogels modified with β-glycerophosphate, for example, undergo sol–gel transitions at body temperature and have been used to deliver chondrogenic agents in situ with minimal invasiveness [33]. Additionally, chitosan derivatives conjugated with methacrylate or aldehyde groups are being explored for dual crosslinking mechanisms, allowing finer control of mechanical and degradation profiles [37].

A growing trend is the convergence of biomaterials with bioelectronics, exemplified by the development of piezoelectric chitosan composites. These materials, which generate localized electric fields upon mechanical stimulation, mimic the electromechanical behavior of native cartilage and bone. In recent preclinical studies, piezoelectric scaffolds based on chitosan/barium titanate exhibited not only enhanced angiogenesis and osteogenesis but also immunomodulatory effects, underscoring their suitability for osteochondral regeneration under dynamic load conditions [30].

Another emerging direction is the use of artificial intelligence (AI) and computational modeling to optimize scaffold design. AI-assisted algorithms can be used to predict the ideal combination of scaffold porosity, degradation kinetics, and mechanical properties to match specific patient anatomy and defect geometry. In silico platforms are also being developed to simulate scaffold–tissue interactions over time, potentially reducing the need for animal models in early validation stages [38]. Furthermore, AI-integrated high-throughput screening can accelerate the discovery of chitosan-based bioinks or composite materials with superior biological performance [39].

Four-dimensional bioprinting, which introduces the dimension of time to conventional 3D printing, represents a novel technique for fabricating dynamically evolving constructs. Chitosan’s tunable structure and biocompatibility make it an attractive candidate for 4D systems that can reshape, self-heal, or respond to cellular signals after implantation [34]. For instance, hybrid chitosan–cellulose-based hydrogels are being engineered to undergo shape-memory transformations that facilitate scaffold deployment via minimally invasive techniques.

Finally, the personalization of scaffolds through patient-derived cells, precision imaging (e.g., MRI-based 3D defect mapping), and custom 3D/4D printing will likely become the standard in the next decade. Incorporating autologous chondrocytes or MSCs into scaffold fabrication—either intraoperatively or pre-loaded ex vivo—will allow tailored tissue constructs with a reduced risk of immune rejection and improved integration.

Taken together, these innovations signal a shift from static, off-the-shelf biomaterials to dynamic, patient-specific bioactive systems, where chitosan remains a highly adaptable and central material. Collaborative efforts among engineers, material scientists, and clinicians will be essential to realize this vision and ensure the clinical viability of advanced chitosan-based scaffolds in cartilage repair.

## 7. Conclusions

Chitosan-based scaffolds have emerged as a promising class of biomaterials for cartilage regeneration, driven by their intrinsic biocompatibility, biodegradability, structural resemblance to glycosaminoglycans, and remarkable chemical tunability. Over the past decade, substantial advancements have been made in scaffold design, including electrospun architectures, 3D-printed constructs, and composite systems integrating growth factors, nanomaterials, and immunomodulatory agents. These developments have enabled improved control over stem cell differentiation, matrix formation, and host tissue integration—both in vitro and in vivo [5,9,17].

Recent in vivo studies in animal models have demonstrated that chitosan scaffolds can support hyaline-like cartilage formation, modulate immune responses, and facilitate osteochondral healing through mechanisms involving M2 macrophage polarization, angiogenesis, and targeted growth factor release [14,30,33]. Despite these encouraging preclinical results, clinical translation remains in its early stages. As outlined in our review of recent trials, few formulations have advanced into late-stage clinical testing, and further longitudinal data on functional outcomes, scaffold degradation, and long-term safety are needed [40].

Significant barriers to clinical application persist, including batch-to-batch material variability, challenges in regulatory classification, and the complexity of achieving scalable, GMP-compliant manufacturing. However, recent advances in stimuli-responsive hydrogels, piezoelectric materials, and AI-assisted scaffold design offer a clear roadmap toward more intelligent, adaptable, and patient-specific therapeutic platforms [39,40,41,42].

Looking ahead, the successful integration of chitosan-based scaffolds into routine clinical practice will depend on a synergistic approach combining material innovation, translational science, and regulatory strategy. Establishing standardized quality metrics, leveraging computational tools for personalized design, and conducting well-powered clinical trials with validated endpoints will be critical to realizing the full therapeutic potential of this biomaterial.

As the field of cartilage tissue engineering continues to evolve, chitosan-based scaffolds stand poised to serve not only as structural supports but as biologically active, multifunctional platforms capable of transforming regenerative orthopedic care.

## Figures and Tables

**Table 1:** Clinical and advanced preclinical trials initiated in 2020 involving chitosan-based systems for cartilage regeneration.

Study Title	Identifier/Source	Start Date	Planned Completion	Study Objective
Long-Term Efficacy of Carboxymethyl Chitosan (CM-C) in Advanced Knee Osteoarthritis	PMID: 11852378	2022	2024	Evaluation of safety and efficacy of intra-articular CM-C injections in patients with advanced knee osteoarthritis unresponsive to hyaluronic acid.
Clinical and Imaging Evaluation of Chitosan Scaffold in Patellar Cartilage Lesions	PMCID: PMC11193853	2015	2024 (5-year follow-up)	Long-term clinical and MRI-based evaluation of patients with patellar cartilage injuries treated with microfracture surgery assisted by chitosan scaffold.
Novel Chitosan–Peptide Composite for Cartilage Tissue Engineering	DOI: 10.1016/j.biopha.2024.113695	2023	2025	Development of a novel composite biomaterial combining chitosan and a cartilage-stimulating peptide (derived from CPNE protein) for cartilage regeneration.
Immunomodulatory Exosomes Loaded in Methacryloyl Chitosan for Cartilage Repair	DOI: 10.1016/j.matdes.2025.112345	2024	2026	Evaluation of the regenerative potential of exosomes embedded in methacryloyl chitosan, aimed at promoting cartilage repair via immunomodulation.
Injectable Thermoresponsive Chitosan-Based Hydrogel for Osteochondral Regeneration	DOI: 10.1021/acsomega.4c10829	2024	2026	Assessment of an injectable, thermosensitive chitosan hydrogel for osteochondral repair in preclinical animal models.
